# Generation and characterization of a zebrafish knockout model of abcb4, a homolog of the human multidrug efflux transporter P-glycoprotein

**DOI:** 10.21203/rs.3.rs-3192988/v1

**Published:** 2023-07-27

**Authors:** Jinhee Park, Hyosung Kim, Leen alabdalla, Smriti Mishra, Hassane Mchaourab

**Affiliations:** Vanderbilt University

## Abstract

The ATP-binding cassette subfamily B member 1 (ABCB1), encoding a multidrug transporter referred to as P-glycoprotein (Pgp), plays a critical role in the efflux of xenobiotics in humans and is implicated in cancer resistance to chemotherapy. Therefore, developing high throughput animal models to screen for Pgp function and bioavailability of substrates and inhibitors is paramount. Here, we generated and validated a zebrafish knockout line of *abcb4*, a human Pgp transporter homolog. CRISPR/Cas9 genome editing technology was deployed to generate a frameshift mutation in exon 4 of zebrafish *abcb4*. The zebrafish *abcb4* homozygous mutant exhibited elevated accumulation of fluorescent rhodamine 123, a substrate of human Pgp, in the intestine and brain area of embryos. Moreover, *abcb4* knockout embryos were sensitized toward toxic compounds such as doxorubicin and vinblastine compared to the WT zebrafish. Immuno-staining for zebrafish Abcb4 colocalized in the endothelial brain cells of adult zebrafish. Transcriptome profiling using Gene Set Enrichment Analysis (GSEA) uncovered that the ‘cell cycle process,’ ‘mitotic cell cycles,’ and ‘microtubule-based process’ were significantly downregulated in the *abcb4* knockout brain with age. This study establishes and validates the *abcb4* knockout zebrafish as an animal model to study Pgp function *in vivo*. Unexpectedly it reveals a potentially novel role for zebrafish *abcb4* in age-related changes in the brain. The zebrafish lines generated here will provide a platform to aid in the discovery of modulators of Pgp function as well as the characterization of human mutants thereof.

## Introduction

ATP-binding cassette (ABC) transporters are membrane-embedded proteins that actively expend the energy of ATP to efflux substrates to the extracellular space [1, 2]. An essential ABC transporter in humans, P-glycoprotein (Pgp), which is encoded by the gene ABCB1, has been shown to play a crucial role in host detoxification of xenobiotic substances [2, 3], leading to multidrug resistance in tumor cells [4]. Specifically, Pgp is expressed on the apical side of endothelial cells within the blood-brain barrier (BBB) and intestinal epithelium to redirect potential toxins into the bloodstream and gut lumen [5–7]. While Pgp plays a significant role in protecting these tissues under normal conditions, its overexpression in tumor cells has been implicated in the refractory treatment of brain malignancies and metastases by chemotherapeutical agents, and in the oral bioavailability of drugs [8].

The direct role of Pgp in drug resistance in conjunction with other ABC transporters has been previously investigated through a mouse model [8–11]. *Abcb1a* and *Abcb1b* (the mouse homologs of human *ABCB1*) knockout mice displayed higher brain penetration of the Pgp substrate ivermectin, causing severe neurotoxicity and death. [11]. Similarly, these mouse models highlighted the function of Pgp in efflux activities after systemic exposure to substrates [9]. *In vitro* studies using tissues from *Abcb1a* knockout mice reported that Pgp modulates drug permeability in the intestinal epithelium. [12]. Thus, mouse models have provided valuable information regarding the function of Pgp. However, they are expensive to maintain and unsuitable for high-throughput screening or non-invasive imaging.

The zebrafish provides distinct advantages as a model for studying the role of ABC transporters such as Pgp. Zebrafish has a structurally similar endothelial membrane system to higher vertebrates, including humans, in the BBB and the intestinal tract [13–15]. Although synteny analysis found that zebrafish has two Pgp orthologs, *abcb4*, and *abcb5* [16], high-throughput screening of human Pgp substrates characterized Abcb4 as functionally phenocopied to human Pgp [17]. It has been reported that the C219 antibody that recognizes human Pgp cross-reacts with zebrafish Abcb4 and Abcb5 [15]. Therefore, coupling antibody staining with RNAscope techniques was required to observe Abcb4 localization in zebrafish [17]. However, the precise characterization of Abcb4 protein expression in zebrafish using immunohistochemistry was still not feasible due to the lack of antibodies specific to zebrafish Abcb4.

Here, we report the generation of an *abcb4* knockout zebrafish model via CRISPR/Cas9 genome editing technology. These lines exhibited a higher accumulation of rhodamine 123 in the gut epithelium. Additionally, *abcb4* knockout embryos show increased susceptibility in response to human Pgp substrates such as vinblastine and doxorubicin. Taking advantage of the cross-reactivity of the human Pgp antibody F4 with zebrafish *Abcb4*, we demonstrate that zebrafish *Abcb4* is localized in various barrier sites such as brain vasculature, intestinal epithelium, and kidney tubules and ducts. Indeed, elevated rhodamine 123 intensity in the brain area of *abcb4* knockout embryos after intravascular injection suggests that *Abcb4* functions as an efflux pump at the BBB. Transcriptome profiling was performed to investigate the function of *abcb4* in the brain, revealing significantly downregulated cell cycle-related pathways. Taken together, our findings established the *abcb4* mutant zebrafish as an effective model for Pgp studies *in vivo*. These lines will provide a platform to investigate potential inhibitors of Pgp and for functional characterization of human mutants via transgenic expression.

## Results

### Generation and validation of a knockout model of the zebrafish homolog of the human ABCB1

**Figure 1:**
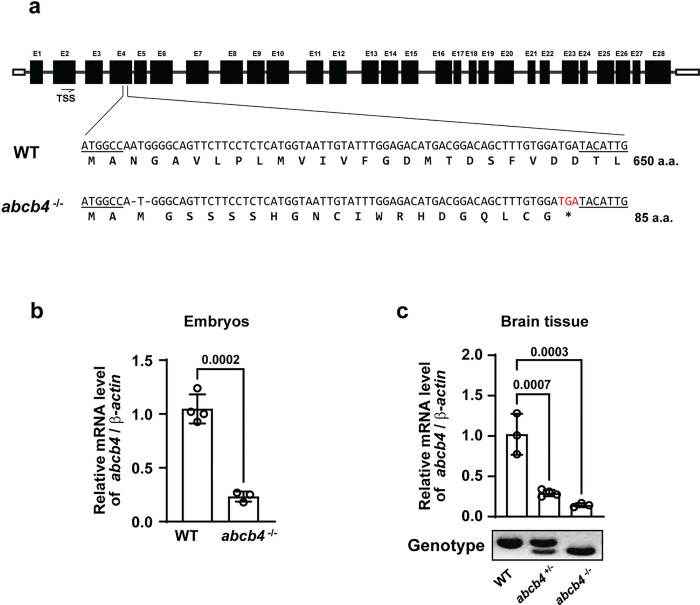
Generation of zebrafish abcb4 knockout mutant using CRISPER/Cas9 system (a) Schematics of the *abcb4* mutant alleles generated using CRISPER/Cas9. The 4^th^ exon of *abcb4* was targeted by gRNA. The sequences of the *abcb4* wild-type (WT) and 2 nucleotides deletion mutant allele (*abcb4*^−/−^) were illustrated. Quantitative RT-PCR showed *abcb4* transcript reduction in the embryos (b) and brain tissue (c) of the *abcb4* mutant. Data are expressed as mean ± SD from at least three independent experiments. P-values were calculated using a two-tailed t-test or one-way ANOVA.

Two orthologs of human ABCB1, *abcb4* and *abcb5*, are found in the zebrafish, of which *abcb4* is functionally similar to human ABCB1 [16, 17]. To elucidate the *in vivo* roles of zebrafish *abcb4* in more details, we generated an *abcb4*-mutated zebrafish line using CRISPR/Cas9 genome editing technology. Guide RNA (gRNA) targeting exon 4 of the genomic sequence of *abcb4* produced a frameshift mutation in the *abcb4* gene leading to a nonsense codon and premature translation termination. Specifically, the mutant allele with a two-nucleotide deletion in exon 4 was predicted to generate truncated Abcb4 proteins of 85 amino acids (Fig. 1a). The *abcb4* transcripts were reduced by 80% in the homozygous knockout embryos, suggesting that the aberrant mRNAs were degraded via nonsense-mediated decay (NMD) (Fig. 1b). In adult zebrafish, the *abcb4* transcripts were reduced 80% in the heterozygous, and 90% in homozygous brain tissues (Fig. 1c). No evident phenotypes were detected in the mutants at the embryonic stages (data not shown).

#### Zebrafish abcb4 knockout lines are defective in the efflux of human Pgp substrates.

**Figure 2:**
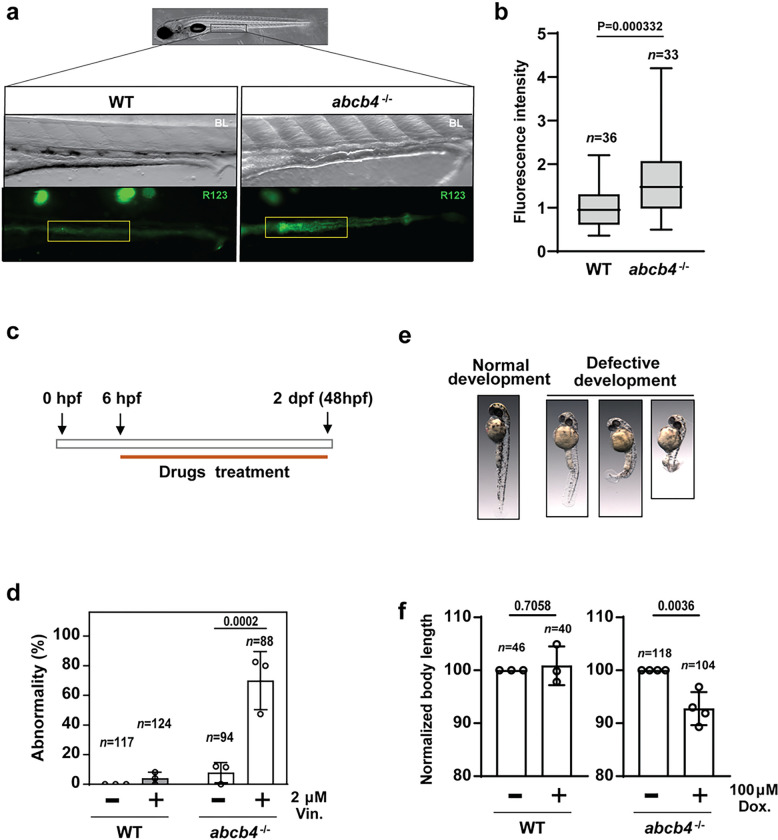
Zebrafish Abcb4 is an efflux transporter of rhodamine123, a substrate for human Pgp (a) Fluorescence micrographs indicate the accumulation rhodamine123 in the WT and *abcb4* knockout embryos. The intensity of rhodamine123 dye in WT and *abcb4* knockout embryos was quantified and illustrated as a bar graph (b). (c) Protocol of drug treatment for vinblastine and doxorubicin toxicity experiments. (e) Representative images of embryos with defects development after vinblastine treatment. (d) The percentage of embryos showing developmental abnormalities for WT and *abcb4* mutant in the presence and absence of 2 mM vinblastine was compared by two-way ANOVA. (e) *abcb4* knockout embryos treated with 100 μM doxorubicin demonstrate a reduction of body length in a two-tailed t-test. Data are expressed as mean ± SD from at least three independent experiments. n numbers indicate the total number of embryos across the independent experiments.

Having demonstrated the reduction of *abcb4* transcripts, we investigated efflux pump activity in the zebrafish *abcb4* mutant using the Human Pgp fluorescent substrate Rhodamine123 as a proxy for efflux transporter function[18]. For this purpose, WT and *abcb4* mutant embryos at 5 days post fertilization (dpf) were incubated in 50 μM of rhodamine 123 for 2 hours, then the level of uptake of rhodamine 123 was examined with fluorescence microscopy. We found higher accumulation of rhodamine 123 in the mid-intestines of the *abcb4* mutant embryo compared to the WT (Fig. 2a). Moreover, WT embryos showed rhodamine 123 fluorescence primarily in the intestinal lumen, whereas *abcb4* mutant embryos displayed the fluorescence in the gut epithelium. To quantitively compare rhodamine123 accumulation in the mid-intestine between WT and *abcb4* mutant, the intensity of its fluorescence was measured and analyzed by Fiji software [19]. The results confirmed that rhodamine123 was significantly higher in the intestine area of the *abcb4* mutant (Fig. 2b).

Having confirmed the reduced efflux of rhodamine123 in *abcb4* knockout embryos, we performed an embryo cytotoxicity assay [21] with other known substrates of human Pgp, vinblastine and doxorubicin [22], to determine to what extent the chemical resistance of the zebrafish embryo is associated with *abcb4* transporter activity. In this assay, embryos were exposed to the compounds 6–48 hours post fertilization (hpf), then developmental defects of embryos, including vertebral malformation and growth retardation, were determined (Fig. 2c and e). We found that developmental abnormalities were highly elevated in *abcb4* mutant embryos in the presence of 2 μM vinblastine compared to WT embryos (Fig. 2d). Toxicity of doxorubicin at 100 μM appears to be low; we did not observe severe developmental malformations in either WT or *abcb4* mutant groups. Indeed, substantially higher concentrations of doxorubicin are required to induce lethal effects [23]. However, *abcb4* deficient embryos displayed greater growth retardation with doxorubicin treatment (Fig. 2f). The overlapping substrate specificity strongly supports *Abcb4* as the functional zebrafish homolog to human Pgp, suggesting our *abcb4* knockout zebrafish is a tractable model for screening Pgp substrates.

#### Organ-specific expression patterns of Abcb4 in adult zebrafish.

**Figure 3:**
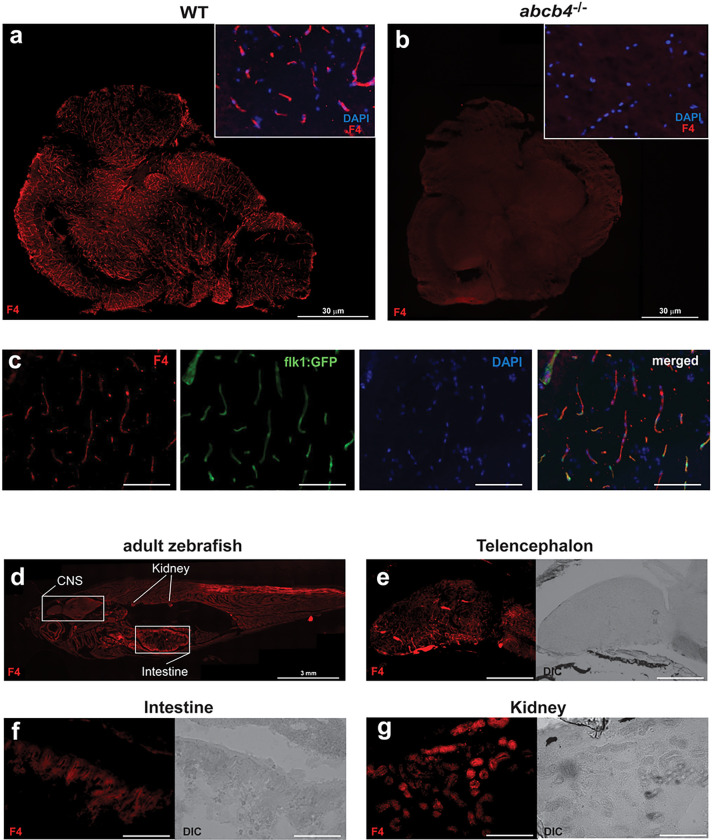
Zebrafish Abcb4 protein localizes to blood vessels in the zebrafish brain. Brain tissues of WT (a) and *abcb4* knockout (b) adult zebrafish, as a negative control, were stained with anti-Pgp antibody F4 (red) as described in the Materials and Method section. Bar =30 mm. (c) F4 antibody staining of whole adult zebrafish. Bar =3 mm. Positive staining (red) was noted in the forebrain (d), intestine (e), and a subset of renal tubes or collecting ducts in the kidney (f). (g) The F4 positive staining (red) in the brain colocalized with flk1:GFP positive cells (green). Fluorescence channels were interrogated individually and merged in. Nuclei were stained with DAPI (blue). Bar =300 mm for (d, e), 200 mm for (f), and 50 mm for (g).

In light of the tissue-specific expression of zebrafish Abcb4, we sought to localize the expression pattern of Abcb4 to determine whether it could be utilized as a model system of the BBB or for oral drug bioavailability screening. It has been reported that the human Pgp C219 antibody cross-reacts with both zebrafish Abcb4 and Abcb5 [13]. Therefore, to identify antibodies that exhibit cross-reactivity with zebrafish Abcb4 proteins, commercial human Pgp antibodies were screened using immunohistochemistry analysis of adult WT and *abcb4* knockout brain tissues. The human Pgp antibody F4 showed zebrafish Abcb4-specific immunoreactivity in the WT adult zebrafish brain but not in *abcb4* knockout tissues (Fig. 3a and b). Higher magnification images indicated that the pattern of F4 positive staining in WT zebrafish likely corresponds to the structure of brain vasculature (Fig. 3a).

To confirm zebrafish Abcb4 expression in the brain vessels, we performed immunostaining of the F4 antibody in the flk1:GFP transgenic line, which expresses GFP in blood vessel endothelial cells. Our staining showed that expression of zebrafish Abcb4 (red) colocalized with flk1:GFP positive endothelial cells (green) throughout the CNS (Fig. 3c). Staining with the F4 antibody of the whole fish (Fig. 3d) revealed positive F4 signal (red) in the renal tubules of the kidney (Fig. 3e) and in the intestinal epithelium (Fig. 3f) as well as the brain (Fig. 3f). This expression pattern is similar to that of human Pgp [24].

#### Probing the function of Abcb4 at the blood-brain barrier of zebrafish.

**Figure 4:**
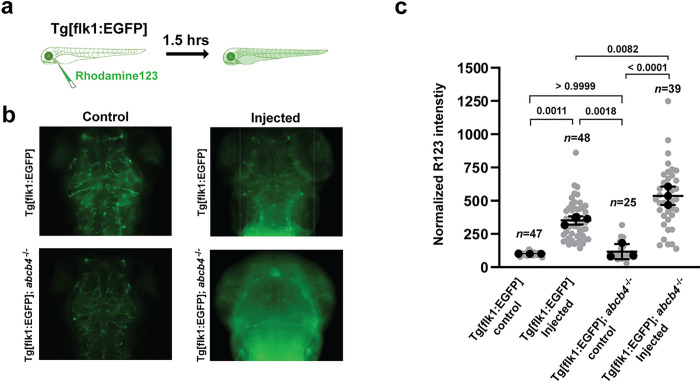
Efflux activity of zebrafish Abcb4 in the larval brain vasculature (a) Diagram of the intravascular injection of rhoadaine123 experiment. Rhodamine123 (green) was injected into the cardinal vein of Tg[flk1:EGFP] and Tg[flk1:EGFP]; *abcb4*^−/−^ embryos at 3 dpf and allowed to circulate for 1.5 hours before imaging. (b) Representative images of the dorsal view of the larval brain after rhodamine123 injection showed the level of rhodamine123 accumulation in the brain area. (c) Quantification of normalized rhodamine123 intensity in the brain area of Tg[flk1:EGFP] and Tg[flk1:EGFP]; *abcb4*^−/−^ embryos. Data are expressed as mean ± SD from three independent experiments (black dots). N numbers (gray dots) indicate the total number of embryos across the three independent experiments. P-values were calculated using two-way ANOVA.

Based on the observation that zebrafish Abcb4 is expressed explicitly in brain vasculature (Fig. 3c), we examined the efflux activity of Abcb4 at the BBB in zebrafish. For this purpose, we performed intracardiac injection of rhodamine123 at 3 dpf embryos of Tg[flk1:EGFP] and Tg[flk1:EGFP];*abcb4*^−/−^ and imaged live fish after 0.5 hours of circulation (Fig. S1). The parenchymal intensity of rhodamine123 dye was elevated ~ 5-fold in both groups compared to non-injected groups, and there was no significant difference between Tg[flk1:EGFP] and Tg[flk1:EGFP];*abcb4*^−/−^ (Fig. S1). This result indicates that the BBB of zebrafish embryo at 3 dpf is permeable, which agrees with previous studies that the BBB is functionally immature at 3 dpf [13, 25]. Interestingly, images of injected embryos after 1.5 hours of circulation showed that the level of rhodamine123 remaining in the parenchyma of Tg[flk1:EGFP];*abcb4*^−/−^ was significantly higher than that of Tg[flk1:EGFP] (Fig. 4b and c). The result suggests that Abcb4 functions as an efflux pump of rhodamine123 in the brain of 3 dpf zebrafish embryos. We note that this finding is in contrast with a previous study that reported the lack of rhodamine123 transport in the brain of 3 dpf zebrafish larvae [13].

#### Age-related changes in the zebrafish brain transcriptome due to loss of abcb4 function.

**Figure 5:**
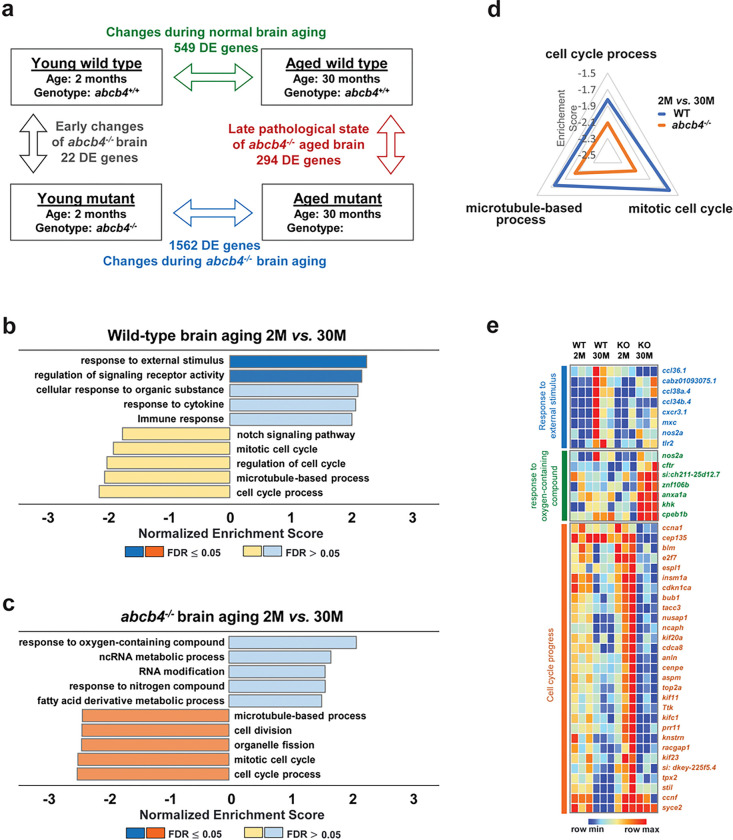
Age-related transcriptome profiling in *abcb4* knockout zebrafish brain (a) Summary of significant DE genes from RNA-seq analysis of brain tissues between WT and *abcb4*knockout zebrafish at 2 and 30 months. Gene Set Enrichment Analysis (GSEA) of age-associated DE genes (FDR < 0.5) in brain tissue of WT (b) and *abcb4* mutant (c) (see Additional file 2 for detailed gene lists of GSEA). (d) Radial graph depicting the three enriched pathways more negatively regulated with age in *abcb4*mutated brain than WT. (e) Heatmaps illustrate the relative level of transcripts in the enriched pathways from GSEA.

Age-associated decline in Pgp function could facilitate the accumulation of toxic substances in the brain, thus increasing the risk of neurodegenerative pathology with aging [10]. To gain insight into the function of Abcb4 in the brain, especially how age-related xenobiotic accumulation alters global molecular regulation, we performed brain transcriptome analysis of WT and *abcb4* knockout at two different age groups. RNA-seq analysis identified Differentially Expressed (DE) genes with FDR cut-off ≤ 0.05 between WT and *abcb4* knockout brain tissues at 2 and 30 months (Fig. 5). At 2 months, there were only 22 DE genes between WT and *abcb4* deficient brains. However, at 30 months, the number of DE genes in brain tissue between WT and *abcb4* knockout fish increased to 294, suggesting that the loss of *abcb4* on the brain transcriptome is aggravated with age (Fig. 5a, see Additional file 1 for DE gene list).

To derive a global understanding of age-related molecular signatures in WT and *abcb4* deficient brains, the DE genes between groups were used for further Gene set enrichment analysis (GSEA) using the WEB-based GEne SeT AnaLysis (WebGestalt) Toolkit (see Additional file 2 for detailed genes lists of GSEA) [26]. In WT, positively enriched categories between 2 and 30 months included ‘response to external stimulus,’ ‘regulation of signaling receptor activity, ‘cellular response to organic substances,’ and ‘cytokine response’ (Fig. 5b, e). Specifically, mRNA level of chemokine ligands such as ccl34b.4 and ccl36.1 were upregulated in WT brain with age. However, these positively regulated pathways in WT with aging were not detected in *abcb4* deficient brain. Conversely, genes associated with oxidative stress are upregulated in the *abcb4* knockout brain but not in WT (Fig. 5c, e). In the age comparisons between 2 and 30 months of WT and *abcb4* knockout brain, both showed ‘mitotic cell cycle,’ ‘cell cycle process,’ and ‘microtubule-based process’ as negatively enriched categories (Fig. 5b, c), which are pathways associated with cell division. Moreover, the three pathways are more significantly down-regulated in the *abcb4* knockout-aged brain than in WT. Thus, more genes involved in the cell division-related pathways are negatively regulated in the *abcb4*-depleted brain with age (Fig. 5e).

## Discussion

The study reported here takes advantage of the power of zebrafish as a model organism to generate the first knockout model of *abcb4*, a functional homolog of human Pgp. In zebrafish, Abcb4 and Abcb5 are both associated with efflux transport activities. However, zebrafish Abcb4 protein has a highly overlapping substrate specificity profile with human Pgp [17]. In addition, previous studies based on morpholino knockdown found that zebrafish Abcb4 transports several fluorescent Pgp substrates in embryos [16]. Our work expands on these previous studies by establishing an *abcb4* knockout animal model. We showed that *abcb4* mutant embryos exhibited a higher accumulation of rhodamine123 in the gastrointestinal tract (Fig. 2a). Interestingly we note that rhodamine123 accumulation in the mid-intestine of *abcb4* knockout embryos overlaps with lysosome-rich enterocytes (LREs) that internalize dietary protein via receptor-mediated and fluid-phase endocytosis for intracellular digestion and trans-cellular transport [20]. This observation could suggest that zebrafish *abcb4* plays a vital function in the lysosomal trafficking of substrates in intestinal cells, although further detailed analysis is needed. In addition to the established role of plasma membrane Pgp, lysosomal Pgp has also been shown to transport cytotoxic agents [27, 28]. Thus, our results suggest a similar role for Abcb4 in the lysosomal membrane of the zebrafish gastrointestinal tract.

We observed high reactivity of the Abcb4-specific antibody F4 in the gastrointestinal tract and renal kidney tubes (Fig. 3e and f), suggesting a high level of Abcb4 expression in the zebrafish gut and kidney. We also noted F4 antibody reactivity in the endothelial cells of the zebrafish brain, indicating that Abcb4 is expressed at the BBB. Moreover, zebrafish *Abcb4* functions as an efflux transporter at 3 dpf embryos (Fig. 4 and Fig. S1). Thus, our findings suggest that the *abcb4* knockout could serve as a powerful zebrafish model, including penetration of drugs at the BBB and pre-clinical examination of oral drug bioavailability and disposition.

A previous human study reported a decrease in Pgp function in the BBB with age [29]. In addition, it has been reported that Pgp deficiency at BBB increases Aβ–deposition in an Alzheimer disease (AD) mouse model [30]. Thus, in mammals, the age-dependent loss of Pgp function may be involved in developing age-related disorders. We re-analyzed a previous zebrafish brain transcriptome and found that the level of *abcb4* transcript is not changed with age [31], which agrees with our RNA-seq data. However, the protein level of zebrafish Abcb4 or the transporter activities of Abcb4 may be modulated with age. Examining the changes in the level of Abcb4 protein in old zebrafish is underway to test this possibility.

The pathway analysis of RNA-seq between WT and *abcb4* knockout brain tissues at different ages brings to the forefront a critical role of Abcb4 and possibly human Pgp in aging. The observation of a negative correlation between cell cycle-related pathways and aging in the transcriptome of WT zebrafish brain suggests that downregulation of the cell cycle-related pathways is part of normal aging, yet it is potentiated in the *abcb4* knockout. Therefore, an important question is whether toxic substances accumulating in the *abcb4* depleted zebrafish brain may cause this further downregulation. Interestingly, it has been suggested that senescence-associated signatures are correlated with increasing aneuploidy and genomic instability due to the downregulation of genes involved in the cell cycle and mitosis progression [32–35]. For example, *cenpe*, one of the core genes encoding protein involved in spindle assembly and chromosome segregation, is downregulated after the onset of senescence [34, 36]. Indeed, the mRNA levels of key players in the cell cycle, including *cenpe* and *aspm,* were dramatically reduced in the brain tissue of the *abcb4* mutant at 30 months compared to WT (Fig. 5e). If so, loss of *abcb4* may play a vital role in inducing an accelerated senescence process in the brain by increasing genome instability, although further experiments are needed to understand the underlying mechanisms.

## Materials and methods

### Zebrafish maintenance and breeding

AB wild-type strain zebrafish (Danio rerio) were used. The embryos were obtained by natural spawning and raised at 28.5°C on a 14:10 h light/dark cycle in egg water 30 mg/L instant ocean in deionized water. Embryos were staged according to their ages (in dpf). All animal procedures were approved by the Vanderbilt University Institutional Animal Care and Use Committee. For *abcb4* knockout genotyping, forward primer 5’-CTTGGC TTAATCATGTCGATGGCCA-3’ and reverse primer 5’-TGTCATCTTCTCCCCCAAAG-3’ were used for PCR amplification. The resulting PCR products were digested with the restriction enzyme NcoI to identify the WT and mutant genotypes.

### Quantitative reverse transcription PCR

Zebrafish were sacrificed, and brain tissues were dissected as described [37]. Tissues were immediately snap-frozen in liquid nitrogen, and RNA was extracted using TRIzol (Invitrogen) and RNA clean & concentrator kit (Zymo Research). 500 μg of total RNA was then used as a template with the SuperScript III First-Strand Synthesis kit (Invitrogen) to produce cDNA. The specific targets were amplified by RT-PCR using oligonucleotides (*abcb4* forward 5’-GCAGGACGTCAGGTGAAGAA-3’, *abcb4* reverse 5’-TGAGTTGTCCCGTCTCGTTG-3’; *b-actin* forward 5’-ACATCCGTAAGGACCTG-3’, *b-actin* reverse 5’-GGTCGTTCGTTTGAATCTC-3’). Samples were analyzed by normalizing expression levels to *b-actin*, and relative quantification was performed using the standard 2-ΔΔCt method.

### Immunostaining

Zebrafish tissues were fixed with 4% paraformaldehyde and processed for immunofluorescence staining. Samples were permeabilized with 0.3% Triton X-100 in PBS, blocked with 10% goat serum in PBS, and incubated at 4°C overnight with the following primary antibodies: mouse anti-Pgp (1:200 dilution, MA5-13854 Invitrogen), goat anti-GFP (1:1000 dilution, 600-141-215, Rockland). The following day, after washing with PBS for unconjugated antibodies, immunostaining was completed by a 1-hour room temperature incubation with secondary antibody (donkey anti-mouse Alexa Fluor 555; 1:1000 dilution; Thermo Fisher Scientific). Tissue sections were mounted with the anti-fade Fluoromount-G medium containing 4’,6-diamidino-2-phenylindole dihydrochloride (DAPI; Southern Biotechnology). Images were acquired with a Leica DMi8 epifluorescence microscope.

### RNA-Seq

Total RNA from zebrafish brain tissues was isolated using TRIzol (Invitrogen) and RNA clean & concentrator kit (Zymo Research). RNA-Seq libraries (n = 3) were processed at the Vanderbilt Technologies for Advanced Genomics (VANTAGE) core. Samples were prepared for sequencing using the TruSeq RNA sample prep kit (Illumina) to prepare cDNA libraries after Poly(A) selection. Raw sequencing reads were obtained for the paired-end samples. FASTQ reads were mapped to the zebrafish genome (GRCz11) by HISAT2 (2.2.0). EdgeR (3.30.3) packages were used to measure Differential Expressed (DE) genes that achieved a count per million mapped reads (CPM). Any genes not considered to be detected (CPM < 4) were removed. False Discovery Rate (FDR < 0.05) was utilized for functional enrichment analysis with the WEB-based Gene SeT AnaLysis Toolkit (WebGestalt) [26].

### Measurement of efflux transporter activity in embryos with rhodamine123 fluorescent dye

10 embryos at 5 dpf were placed in one well of a 24-well plate (polystyrene, tissue culture grade) and incubated with 1 ml of 50 μM rhodamine123 (Invitrogen, R302) diluted in 0.3x Danieau water for 2 hours in the dark and rinsed three times with 0.3x Danieau water to remove excess dye. The amount of rhodamine123 accumulated in the gut area of zebrafish embryos was analyzed by fluorescence microscopy (Zeiss Axiozoom V16). Quantification of the intensity of rhodamine123 in the intestine area was performed by the software package Fiji [19]

### Embryotoxicity experiments

For determining the toxicities of vinblastine (Signa, V1377) and doxorubicin (Sigma, D1515), 10 embryos were incubated in a 24 well plate with 1 mL test solutions from 6 hpf until 48 dpf to examine developmental abnormalities. A final abnormality count was performed at 48 hours, and embryos were declared abnormal if at least one of the following criteria applied: i) shortened body length, ii) tail or body curvature. Controls contained DMSO used as a solvent.

### Intravenous microinjections of Rhodamine123

Embryos of Tg[flk1:EGFP] and Tg[flk1:EGFP]; *abcb4*^−/−^ at 3 dpf were immobilized with tricaine and placed in an agarose injection mold. Next, 1 nl of 2 mg/ml Rhodamine123 (Invitrogen, R302) was injected into the cardinal vein of embryos using a standard zebrafish microinjection apparatus. After 1.5 hours of circulation, the brain area of embryos was imaged using fluorescence microscopy (Zeiss Axiozoom V16). For quantification of rhodamine123 intensity in the brain, the green fluorescent signal outside of the vasculature of the larval brains was analyzed by Fiji [19]. Each group’s measured rhodamine123 intensity values were normalized to the basal level of green fluorescent intensity outside of vasculature of non-injected Tg[flk1:EGFP].

### Statistics

Statistical analyses were carried out with GraphPad Prism software (GraphPad) utilizing Student t-test or ANOVA. Comparisons between groups were performed with the Bonferroni test. Statistical significance was defined as P < 0.05

## Data Availability

RNA-seq data have been deposited in the ArrayExpress database at EMBL-EBI (www.ebi.ac.uk/arrayexpress) under accession number E-MTAB-12901.

## The abbreviations used are:

hpf: hours post-fertilization
dpf: days post-fertilization
qRT: quantitative reverse Transcription
PTU: 1-phenyl-2-thiourea
Dox: doxorubicin
Vin: vinblastine
RNA-seq: RNA-sequencing
DE genes: Differential Expressed genes
NMD: nonsense-mediated decay
